# Effects of the Replacement of Dietary Fishmeal by the Blend of *Tenebrio molitor* Meal, *Chlorella* Meal, *Clostridium Autoethanogenum* Protein, and Cottonseed Protein Concentrate on Growth, Protein Utilization, and Intestinal Health of Gibel Carp (*Carassius gibelio*, CAS Ⅴ)

**DOI:** 10.1155/2024/5019899

**Published:** 2024-03-25

**Authors:** Yongning Yu, Xing Wang, Junyan Jin, Dong Han, Xiaoming Zhu, Haokun Liu, Zhimin Zhang, Yunxia Yang, Shouqi Xie

**Affiliations:** ^1^State Key Laboratory of Fresh Water Ecology and Biotechnology, Institute of Hydrobiology, Chinese Academy of Sciences, Wuhan 430072, China; ^2^University of Chinese Academy of Sciences, Beijing 100049, China; ^3^Hubei Engineering Research Center for Aquatic Animal Nutrition and Feed, Wuhan 430072, China; ^4^The Innovative Academy of Seed Design, Chinese Academy of Sciences, Wuhan 430072, China

## Abstract

The trial was conducted to investigate the effects of the replacement of dietary fishmeal (FM) by the blend of *Tenebrio molitor* meal (TMM), *Chlorella* meal (CM), *Clostridium autoethanogenum* protein (CAP), cottonseed protein concentrate (CPC) on growth, protein utilization and intestinal health of gibel carp (*Carassius gibelio*, CAS Ⅴ). The FM-based diet was used as the control, and the blended proteins (TMM: CM: CAP: CPC) at ratios of 1 : 1:8 : 2 (BLEND A), 1 : 1:6 : 4 (BLEND B), and 1 : 1:4 : 6 (BLEND C) were used to replace FM at three levels (33%, 67%, 100%), respectively. The results showed that, compared to the control group, growth performance increased significantly when dietary FM was fully replaced by BLEND B (*P* < 0.05), while decreased by BLEND A (*P* < 0.05). The complete substitution of FM with BLEND B significantly upregulated the mRNA expression of intestinal proinflammatory cytokines, anti-inflammatory cytokines, and tight junction-related genes (*P* < 0.05), improving intestinal tissue morphology and health. And it also significantly increased intestinal trypsin activity (*P* < 0.05), upregulated the mRNA expression of amino acid sensory receptor-related and amino acid or peptide transport-related genes (*P* < 0.05), increased protein apparent digestibility coefficient (*P* < 0.05). The 100% substitution of FM with BLEND A significantly upregulated the mRNA expression of intestinal proinflammatory cytokines and downregulated the mRNA expression of anti-inflammatory cytokine *il-10* (*P*  < 0.05), reduced intestinal villus height (*P* < 0.05), and decreased protein apparent digestibility coefficient (*P* < 0.05). In conclusion, BLEND B could completely substitute dietary FM and was beneficial to the growth and health of gibel carp. Dietary digestible essential amino acids index (DEEAI) was found as an important indicator and should be higher than 79.5% to meet the maximum growth of fish.

## 1. Introduction

Global aquaculture is developing more rapidly than any other food-producing sector, and provides almost one-third of animal protein for human beings [1]. Fishmeal (FM) has been considered as the best protein source for aquafeeds because of its excellent properties [2, 3]. However, FM production cannot meet the requirement of aquaculture due to limited resources [1]. Thus, numerous researches have been conducted on FM substitution in aquafeeds. Many researches have been focusing on a single protein source (e.g., soybean protein) to replace FM, which came with the drawbacks of antinutrient factors and unbalanced amino acid composition that limit the replacement level of FM [4–6]. On the other hand, soy is also used for human food and requires more land for large production. Therefore, it should be considered to search the new feed ingredients of nonfood proteins [7].

A novel bacterial protein, *Clostridium autoethanogenum* protein (CAP) is extracted and processed from the by-products of fermentation by *C. autoethanogenum* to produce ethanol from industrial waste gas CO [8]. Cottonseed protein concentrate (CPC), is a nonfood plant protein, adopted by low-temperature drying and solvent extraction, effective reduction of the thermal denaturation of protein, and removement of free gossypol [9]. *Tenebrio molitor* meal (TMM) is an insect protein from the kitchen waste [10, 11]. *Chlorella* meal (CM) is a single-cell algae of high productivity, and high contents of valuable constituents [12]. It has been reported that these four proteins are all rich in protein and have good amino acid composition [9, 13–15]. Wu et al. [16] found that a single protein of CAP could replace dietary FM up to 30% without adverse effects on growth performance in carnivorous large yellow croaker (*Larimichthys crocea*). However, 45% replacement reduced the activity of intestinal protease, damaged intestinal health, and inhibited growth. CAP also found that optimal FM replacement was 14.55% in omnivorous black sea bream (*Acanthopagrus schlegelii*), while the minimum weight gain was observed in 58.2% FM replacement [13]. In omnivorous white shrimp (*Litopenaeus vannamei*), the dietary 30% FM replacement by CAP had no adverse effects on growth performance or intestine health, while the dietary 45% FM replacement showed negative effects on fish [17]. CPC was reported to be acceptable to replace 50% of dietary FM in carnivorous largemouth bass (*Micropterus salmoides*), while negative effects were observed on intestinal tissue structure, protein utilization, and growth performance at 75% replacement [18]. CPC, as a single protein source, could only replace 48% dietary FM in omnivorous golden pompano (*Trachinotus ovatus*) [19] or 40% dietary FM in omnivorous white shrimp (*L. vannamei*) [20] without negative effects on growth performance. CM contains *Chlorella* growth factors and TMM contains chitin. *Chlorella* growth factor and chitin have positive effects on feeding, growth, and immunity of fish, while high chitin has antinutritional effects [12, 15, 21]. The limited replacement level might be due to unbalanced amino acid composition that could not achieve the aquaculture animal requirements. Therefore, it is necessary to consider an optimal blend of TMM, CM, CAP, and CPC so as to increase the replacement level of FM in aquafeeds.

Gibel carp (*Carassius gibelio*) is an important freshwater aquaculture fish, and its production in China was higher than 2.85 million tons in 2022 [22]. As an omnivorous fish, gibel carp was also found to show better growth when fed with high-FM feed [4, 5]. The present study was designed to use blends of TMM, CM, CAP, and CPC of different ratios to replace different levels of dietary FM so as to find the optimal blend and replacement level when considering growth, protein utilization, and intestinal health.

## 2. Materials and Methods

### 2.1. Experimental Diets

The formulation and chemical composition of experimental diets are presented in Table 1. The amino acid composition is shown in Table 2. During the diet preparation, TMM, CM, CAP, and CPC first were grounded respectively, and then mixed at different proportions to prepare three blended proteins (TMM: CM: CAP: CPC) at ratios of 1 : 1:8 : 2 (BLEND A), 1 : 1:6 : 4 (BLEND B), and 1 : 1:4 : 6 (BLEND C). The control diet was formulated with 15% FM, while the blended proteins were used to replace 33%, 67%, and 100% FM, respectively, to prepare ten isonitrogenous (34.20% crude protein) and isolipidic (7.60% crude lipid) practical diets (Control, A33, A67, A100, B33, B67, B100, C33, C67, and C100). All ingredients were finely ground to less than 150 *μ*m with the laboratory machine (JYNU30-18.5M, Jeinna Co., Ltd.) and then thoroughly mixed and pelleted (SLP-45; Fishery Machinery and Instrument Research Institute, Chinese Academy of Fishery Sciences, Shanghai, China). The pellets were oven-dried at 60°C and stored at 4°C for later use.

### 2.2. Feeding Trial

Gibel carp used in this study were collected from the Guanqiao hatchery farm of the Institute of Hydrobiology, Chines Academy of Sciences (Wuhan, China). The experiment was carried out in net cages (2.0 m × 2.0 m, water depth 1.8 m) located at the center of the pond (29°50′21.372″N, 112°28′39.504″E, Shishou Original Seed Stock Farm of Four Major Carps, Jingzhou, Hubei, China). All fish were fed with commercial diets (103, Tongwei Co., Ltd.) 3 meals/day for 2 weeks for acclimation. At the beginning of the trail, fish of similar size (weight: 15.18 ± 0.20 g) were selected, bulk-weighed, and randomly distributed into 30 cages at a density of 70 fish per cage. Subsequently, the fish were randomly allocated to ten diets with three cages per treatment. During the trial, gibel carp were fed up to apparent satiation at 7 : 00, 12 : 00, and 17 : 00 for 8 weeks (from 1.7.2021 to 28.8.2021). The water around cages was aerated at night for 10 hr from 8 : 00 p.m. to 6 : 00 a.m. The natural light and temperature were used. The water temperature (0.5 m under the water surface) was monitored every day and was 29.2–34.0°C. The water ammonia nitrogen was below 0.4 mg L^−1^, pH was 7.4–7.6, and dissolved oxygen was higher than 5 mg L^−1^.

### 2.3. Sample Collection

At the end of 8-week feeding trial, fish were fasted for 12 hr. All fish in each cage were anesthetized with MS-222 (50 mg kg^−1^; Sigma, USA) and bulk-weighed. Two fish per cage were collected for proximate composition, another three fish for the calculation of body index, and another two fish for plasma, liver tissues, and intestinal tissues. The liver tissues were used for transcriptome sequencing, and the intestinal tissues were used for enzyme activity, real-time polymerase chain reaction (RT-PCR), paraffin section, and electron microscope section.

### 2.4. Digestibility Determination

After the feeding trial, the remained fish after sampling were transferred into a recirculating aquaculture system (Wuhan, China), and fed with the corresponding experimental diets for digestibility determination. Fish were reared in fiberglass tanks (diameter 1.5 m, water depth 0.60 m). The water temperature (0.5 m under the water surface) was 27.9–30.1°C. Light period was from 8 : 00 to 20 : 00. The water ammonia nitrogen was below 0.2 mg L^−1^, the pH was 7.0–7.2, and the dissolved oxygen was higher than 7 mg L^−1^. The fish were fed three meal per day (7 : 00, 12 : 00, and 17 : 00) and the uneaten feed were collected after each feeding. The fresh and intact feces were collected by siphoning 4 hr after feeding from the 2nd week. Then, feces were freeze-dried and stored at −20°C for subsequent analysis. The yttrium trioxide was as an indirect indicator for the calculation of apparent digestibility.

### 2.5. Biochemical Analysis

The proximate composition of diets, fish and feces were analyzed in accordance with the Association of Official Analytical Chemists (AOAC) [28] method. Moisture, ash, crude protein, crude lipid, amino acid contents, and yttrium trioxide contents were determined using the methods described by Liu et al. [5]. Gross energy was assessed using a Philips Microbomb Calorimeter (Gentry Instruments Inc., USA).

According to the manufacturer's instructions, the measurement of triglycerides (TG), cholesterol (CHO), and glucose (GLU) in plasma was conducted using commercial assay kits (290–63,701, 294−65,801, 298–65,701; Wako Pure Chemicals, Japan), and the plasma free amino acid (FAA) measurement was conducted using commercial assay kits (A026-1-1; Nanjing Jiancheng Bioengineering Institute, Nanjing, China).

### 2.6. Quantitative Real-Time PCR Analysis

The Trizol (Invitrogen, USA) was utilized to extract total RNA from the intestine tissues following the manufacturer's instructions, then agarose gel electrophoresis was utilized to detect RNA integrity. The NanoDrop® Spectrophotometer (NanoDrop Technologies, USA) was utilized to determine RNA concentration. The Moloney murine leukemia virus (M-MLV) FirstStrand Synthesis Kit (Invitrogen, Shanghai, China) was used for reverse transcription of the total RNA. The NCBI primer BLAST service was utilized to design the quantitative polymerase chain reaction (qPCR) primers which were listed in Table 3. *β*-actin was used as the internal reference for normalization. qRT-PCR was conducted on a LightCycle 480 II system (Roche, Switzerland). More details were described by Liu et al. [18]. The relative expression was calculated according to Vandesompele et al. [29].

### 2.7. Intestinal Tissue Section

The intestine tissues stored at paraformaldehyde solution were analyzed by hematoxylin and eosin (H&E) stained sections, and these stored with glutaraldehyde solution were observed under a transmission electron microscope. More detailed steps were described by Liu et al. [30].

### 2.8. Liver Transcriptome Analysis

According to the manufacturer's instructions, Trizol (Invitrogen, USA) was utilized to extract total RNA from the liver tissues. Subsequently, Oligo(dT)-attached magnetic beads were utilized to purify mRNA. Sequencing data were filtered with SOAPnuke, and clean reads were mapped to the reference genome using HISAT2. The gene expression was calculated by RNA-Seq by Expectation–Maximization (RSEM) and differential expression analysis was performed by DESeq2 with a *Q* value ≤ 0.05. GO and KEGG enrichment analyses were performed by Phyper.

### 2.9. Enzyme Activities

The activities of trypsin (TRY) and chymotrypsin (CT) in the intestines were determined using the methods described by Liu et al. [30]. Na-benzoyl-L-arginine 4-nitroanilide hydrochloride (B4875; Sigma, USA) and N-succinyl-ala-ala-pro-phe p-nitroanilide (S7388; Sigma, USA) were used as special substrates for TRY and CT, respectively. The protein concentration of the intestines was determined with a protein assay kit (A045-2; Nanjing Jiancheng Bioengineering Institute, Nanjing, China). Enzyme-specific activity was expressed as units per gram of soluble protein.

### 2.10. Statistical Analysis

All data statistical analyses were performed using SPSS 26.0 for one-way analysis of variance after the homogenesis test. Duncan's multiple tests were applied with a significance level of *P* < 0.05. Data were presented as mean ± standard error (SE). Regression analysis was performed with Graph Pad Prism version 9.1.1.

## 3. Results

### 3.1. Growth Performance

Table 4 showed that there were no significant differences in survival and feeding rate between different treatments (*P* > 0.05). Lower final body weight (FBW), specific growth rate (SGR), feed efficiency (FE), protein efficiency ratio (PER), and protein retention efficiency (PRE) were observed in the fish fed A100 diet (*P* < 0.05), while higher SGR was observed in the fish fed B100 diet than those in the fish fed control diet (*P* < 0.05). Higher FE, PER, and PRE were observed in the fish fed A33 and B100 diets than those in the fish fed control diet (*P* < 0.05).

Table 5 showed no significant differences between groups in condition factor, visceral somatic index, and whole-body composition (*P* > 0.05). Higher hepatosomatic index (HSI) was observed in the fish fed A33 diet than those in the fish fed control, A67, A100, and C67 diets (*P* < 0.05).

### 3.2. Biochemical Index

Table 6 showed that dietary treatments did not significantly affect plasma TG content (*P* > 0.05). The plasma GLU content in the B33 and C groups was lower than that in the control group (*P* < 0.05). The plasma FAA content in the A100 group was significantly lower, while those in the A33 and B67 groups were higher than that in the control group (*P* < 0.05). The plasma CHO content in the C33 group was higher than those in the A100, B67, and C67 groups (*P* < 0.05).

### 3.3. Apparent Digestibility Coefficient (ADC)

Table 7 showed that ADCs of dry matter (ADC_DM_), gross energy (ADC_GE_), crude protein (ADC_CP_), total essential amino acids (ADC_TEAA_), and total nonessential amino acids (ADC_TNEAA_) in the A67 and A100 groups were significantly lower than those in the control group (*P* < 0.05). The ADC_CP_, ADC_TEAA_, and ADC_TNEAA_ in the A33 and B100 groups were significantly higher than those in the control group (*P* < 0.05). Compared to the control group, the ADC of EAAs (leucine, valine, threonine, arginine) and NEAAs (asparagine, glutamic acid, glycine, alanine, tyrosine, proline, cysteine) in the A100 group were significantly decreased (*P* < 0.05). However, the ADC of EAAs (lysine, methionine, threonine, leucine, isoleucine, valine, phenylalanine, histidine) and all NEAAs in the B100 group were significantly increased (*P* < 0.05).

**Figure 1:**
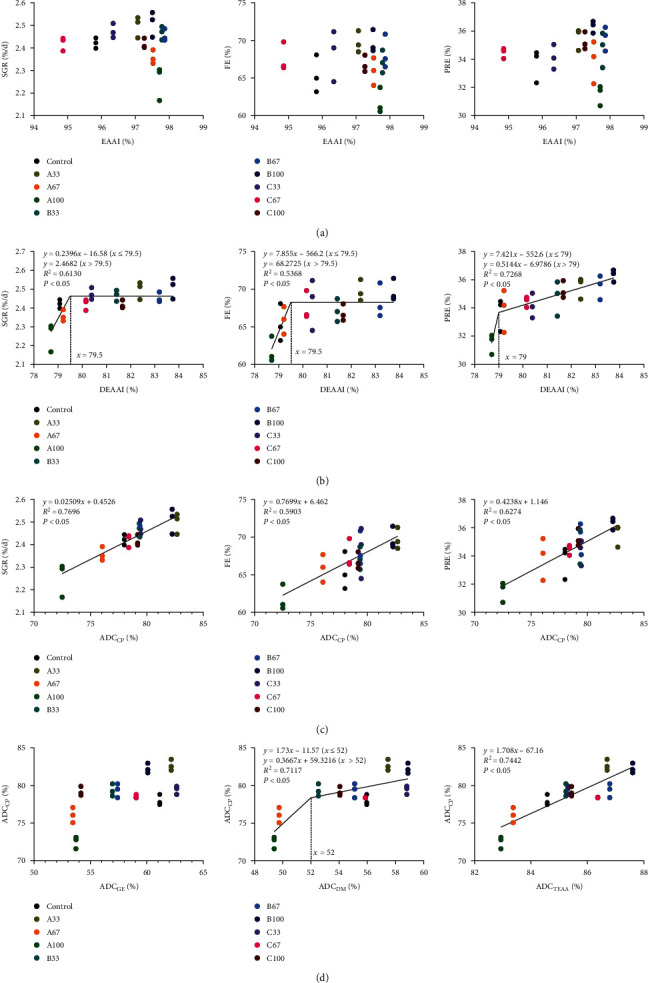
Scatter diagram between essential amino acid index (EAAI) of feed and specific growth rate (SGR), feed efficiency (FE), and protein retention efficiency (PRE) in gibel carp (a). Nonlinear regression relationship between digestible essential amino acid index (DEAAI) and SGR, FE, and PRE (b). Simple linear regression relationship between apparent digestibility coefficient of crude protein (ADC_CP_) and SGR, FE, and PRE (c). Simple linear or nonlinear regression relationship between apparent digestibility coefficients of gross energy (ADC_GE_), dry matter (ADC_DM_), and total essential amino acid (ADC_TEAA_) and ADC_CP_ (d).

Figure 1 showed that there was no significant relationship between SGR, FE, and PRE and the dietary essential amino acid index (EAAI). With the increase of dietary digestible essential amino acid index (DEAAI), SGR and FE increased significantly (reaching the maximum value when DEAAI was 79.5%; *P* < 0.05) and then tended to be stable, while PRE increased significantly (increasing slowly after DEAAI reached 79%; *P* < 0.05). With the increase of ADC_CP_, SGR, FE, and PRE increased significantly (*P* < 0.05). With the increase of ADC_DM_, ADC_CP_ increased significantly (increasing slowly after ADC_DM_ reached 52%; *P* < 0.05). With the increase of ADC_TEAA_, ADC_CP_ increased significantly (*P* < 0.05). There was no significant relationship between ADC_GE_ and ADC_CP_.

**Figure 2:**
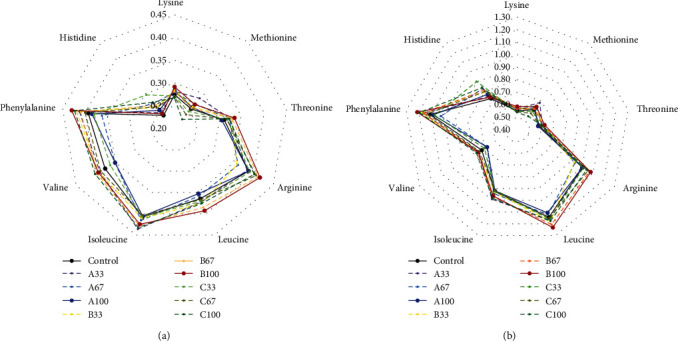
Ratios of dietary digestible essential amino acids (DEAA) to muscle EAA composition (a) and dietary EAA requirements (b).

Digestible essential amino acids are bioavailable for fish.  Figure 2 showed that the ratios of dietary DEAAs to muscle EAAs composition and EAA requirements in the B100 group were higher in most essential amino acids and closer to the muscle and/or requirements.

### 3.4. Liver Transcriptome Analysis

**Figure 3:**
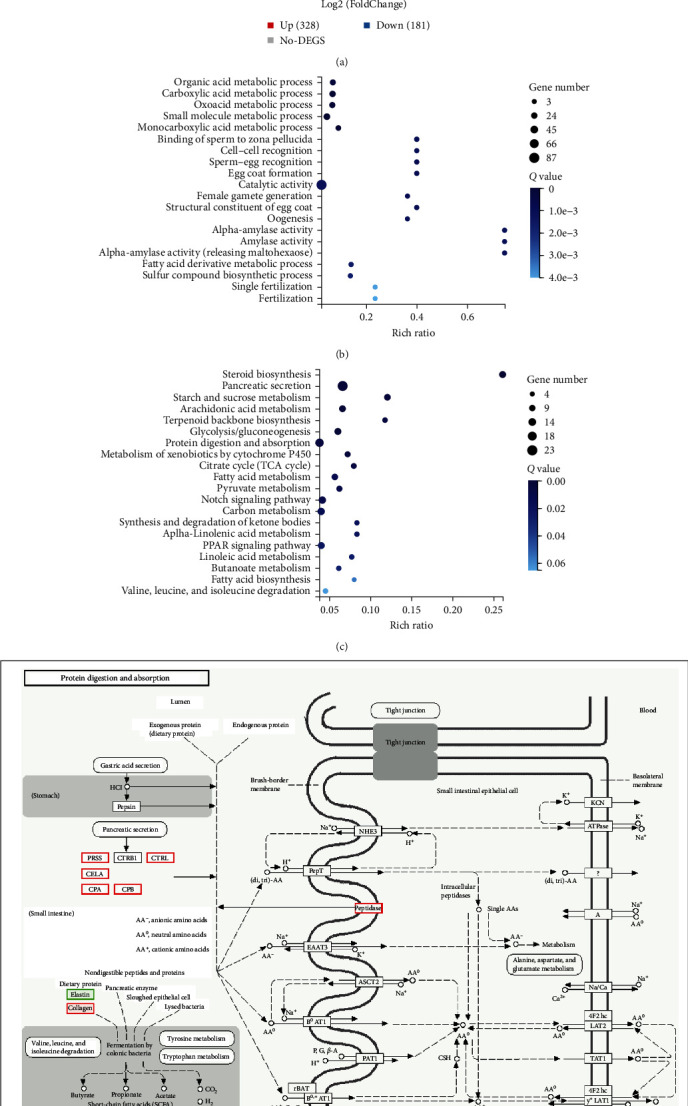
Liver transcriptome analysis in the B100 group compared to the control group: the analysis of differentially expressed genes (a), the enrichment analysis of gene ontology (b) and Kyoto Encyclopedia of Genes and Genomes pathway (c), and the pathway of protein digestion and absorption (d). PRSS, serine protease; CTRL, chymotrypsin-like protease; CELA, cellulase A; CPA, carboxypeptidase A; CPB, carboxypeptidase B.

Figure 3 showed that a total of 328 upregulated differentially expression genes (DEGs) and 181 downregulated DEGs were identified in the B100 group compared with the control group. The main enriched pathways in the B100 group compared to the control group were the organic acid metabolic process, pancreatic secretion, and protein digestion and absorption process. Furthermore, the gene expression of various digestive enzymes in the B100 group was enhanced, including serine protease, chymotrypsin-like protease, cellulase A, carboxypeptidase A, and carboxypeptidase B.

### 3.5. Protein Digestion and Absorption

**Figure 4:**
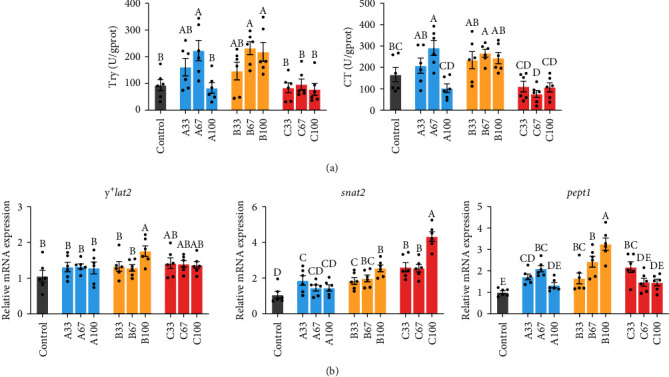
The trypsin and chymotrypsin activity (a), the relative mRNA expression of amino acid or peptide transporter-related genes (b) in the intestine of gibel carp fed the experimental diets. Results are expressed as means ± SE (*n* = 6), and different letters above a bar denote the significant difference between treatments (*P* < 0.05).

Figure 4(a) showed that the activities of intestinal TRY in gibel carp of the A67, B67, and B100 groups increased significantly, as well as the activities of intestinal CT of the A67 and B67 groups compared to the control group (*P* < 0.05).

Figure 4(b) showed that the intestinal amino acid or peptide transporter-related gene expression was affected in the fish-fed blended proteins. Compared to the control group, the mRNA expression of intestinal *y*^*+*^*lat2* of the B100 group, *snat2* of other groups (excluding the A67 and A100 groups), and *pept1* of the A33, A67, B, and C33 groups was significantly upregulated (*P* < 0.05).

**Figure 5:**
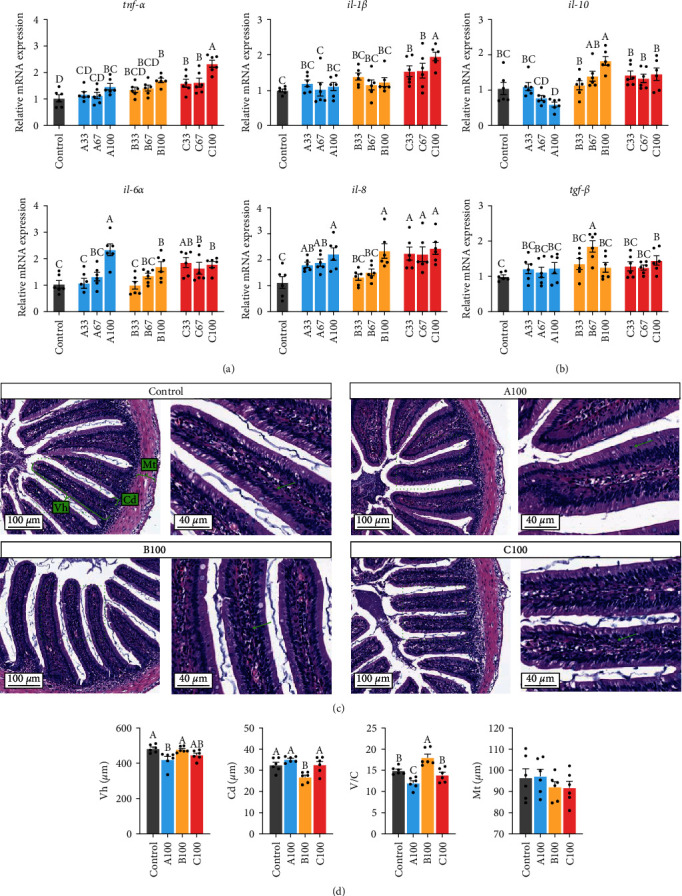
The relative mRNA expression of proinflammatory cytokine (a) and anti-inflammatory cytokine (b) in the intestine of gibel carp fed the experimental diets. The intestine of the control, A100, B100, and C100 groups showed as the H&E staining image (c). The villus height (Vh), crypt depth (Cd), the ratio of villus height to crypt depth (V/C), and muscle thickness (Mt) in intestinal tissue structure of gibel carp in the control, A100, B100, and C100 groups (d). Results are expressed as means ± SE (*n* = 6), and different letters above a bar denote the significant difference between treatments (*P* < 0.05). The right image of each group is the enlarged image inside the dotted box on the left image. The green brackets represent the measurement of Vh, Cd, and Mt respectively. The green arrow indicates lymphocyte.

**Figure 6:**
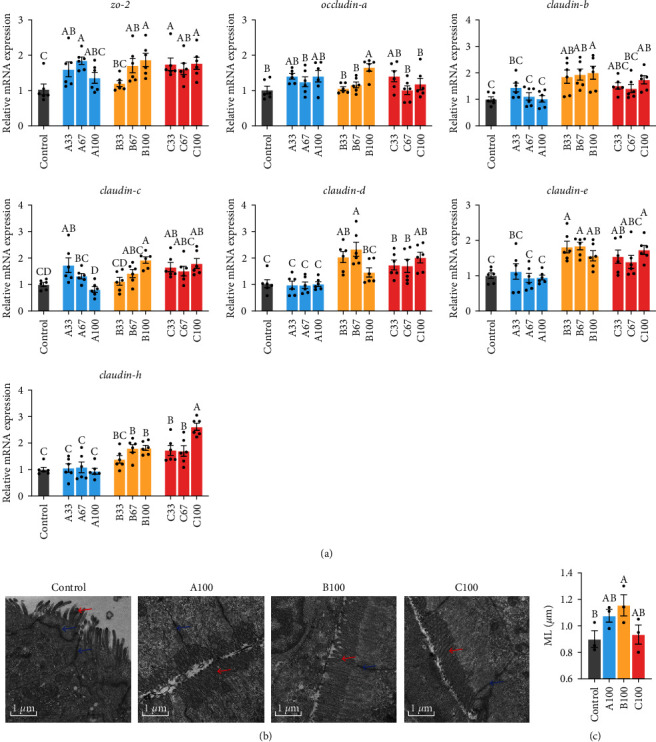
The relative mRNA expression of tight junction-related genes in the intestine of gibel carp fed the experimental diets (a). The intestine of the control, A100, B100, and C100 groups showing as electron microscope images (b). The microvillus length (ML) in the intestinal tissue structure of gibel carp in the control, A100, B100, and C100 groups (c). Results are expressed as means ± SE (*n* = 3 for (c), *n* = 6 for (a)), and different letters above a bar denote the significant difference between treatments (*P* < 0.05). The red arrow indicates Microvilli; blue arrow indicates tight junction.

Figure 5(d) showed that the different dietary did not significantly affect the muscle thickness of fish intestine (*P* > 0.05). The villus height (Vh) and the ratio of villus height to crypt depth (V/C) of the A100 group were significantly lower than those of the control group (*P* < 0.05). The crypt depth (Cd) of the B100 group was significantly lower, and the V/C of the B100 group was significantly higher than those of the control group (*P* < 0.05).  Figure 6(c) showed that the microvillus length (ML) of the B100 group was significantly higher than that of the control group (*P* < 0.05).

### 3.6. Intestinal Health

Figure 5 showed that multiple proinflammatory genes mRNA expression in the other groups was significantly upregulated compared to the control group (*P* < 0.05), including *tnf-α* and *il-6α* in the A100, B100, and C groups, *il-1β* in the C groups, and *il-8* in the A, B100, and C groups. The mRNA expression of anti-inflammatory *il-10* in the B100 group and *tgf-β* in the B67 and C100 groups was significantly upregulated compared to the control group (*P* < 0.05), while the mRNA expression of *il-10* in the A100 group was significantly downregulated (*P* < 0.05). The fish intestinal H&E staining images showed that there were many lymphocytes infiltrating epithelial cells in the intestinal tissue structure of the A100 group.

Figure 6 showed that the substitution of dietary FM by blended proteins upregulated the mRNA expression of multiple intestinal tight junction-related genes (*P* < 0.05), including *zo-2* in the A33, A67, B67, B100, and C groups; *occludin-a* in the B100 group, *claudin-b* in the B and C100 groups; *claudin-c* in the A33, B100, C33, and C100 groups *claudin-d* in the B33, B67, and C groups; *claudin-e* in the B, C33, and C100 groups; *claudin-h* in the B67, B100, and C groups. In addition, the hollowing and severe curvature of the intestinal tight junction was observed in the control group through the intestinal electron microscope images, while no similar phenomenon appeared in the A100, B100, and C100 groups (Figure 6(b)).

### 3.7. Intestinal Amino Acid Sensory Receptor-Related Genes mRNA Expression

**Figure 7:**
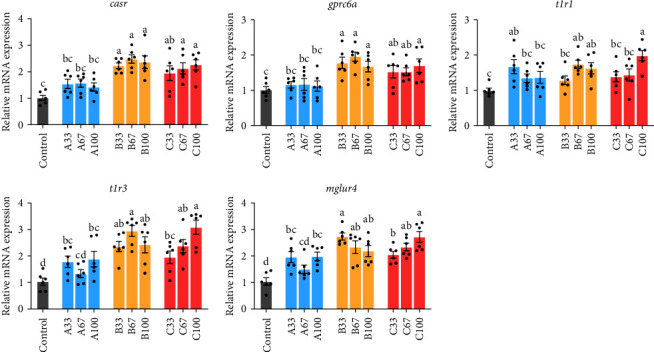
The relative mRNA expression of intestinal amino acid sensory receptor-related genes of gibel carp fed the experimental diets. Results are expressed as means ± SE (*n* = 6), and different letters above a bar denote the significant difference between treatments (*P* < 0.05).

Figure 7 showed that the mRNA expression of intestinal *casr* and *gprc6* in the B and C groups were significantly upregulated compared to the control group (*P* < 0.05). The mRNA expression of intestinal *t1r1* in the A33, B67, B100, and C100 groups was significantly upregulated compared to the control group (*P* < 0.05). The mRNA expression of intestinal *t1r3* and *mglur4* in the A33, A100, B, and C groups were significantly upregulated compared to the control group (*P* < 0.05).

## 4. Discussion

Due to the numerous deficiencies of a single protein source, there has been a growing emphasis on multiple protein source blends as substitutes for FM in the diets for aquaculture [31, 32]. In the present study, dietary BLEND B could fully replace FM and showed even better growth. Better growth performance was reported in many aquaculture species to be achieved by dietary balanced amino acids of mixed proteins compared to single alternative protein [33–35]. On the other hand, the present study also showed that BLEND A was found to obtain poor growth performance at the substitution of 100% dietary FM. Similar results were also reported in rainbow trout (*Oncorhynchus mykiss*) [36] and totoaba (*Totoaba macdonaldi*) with dietary improper blended proteins [32]. These suggested that the unsuitable mixed proteins could not meet the growth requirements of fish.

Normally, it is considered that dietary EAAI is important of the protein quality [37]. In the present study, the growth performance of different diets was different though similar dietary EAAI were composed. Dietary protein quality, which is depended on the AA composition and digestibility, is the dominating factor affecting growth performance of fish [38]. The present study showed that the EAA composition of the blended proteins diets was similar to that of the FM diet, and there was no significant relationship between fish growth and dietary EAA composition. Therefore, dietary EAA composition was not the most important factor affecting the growth of gibel carp. However, a significant positive correlation was found between fish growth and ADC_CP_. Similar results were found in largemouth bass (*M. salmoides*) [39]. It was also found that ADC_CP_ was closely related to ADC_TEAA_. And a positive correlation between fish growth and dietary DEAAI was observed in the present study. The ideal protein model of the feed is closely related to the muscle or whole-body EAA profile of the fish [40, 41]. The present study found that the best growth was achieved at BLEND B which could replace 100% dietary FM. The DEAA of this blended protein was closer to muscle and requirement of EAA composition. The present study found that dietary DEAAI should be higher than 79.5% for maximum growth. It suggested, not only the chemical composition of EAAI, but also DEAAI is important for fish growth.

The liver transcriptome analysis showed that the main enriched pathways were the pancreatic secretion and protein digestion and absorption processes when dietary FM was 100% replaced by BLEND B protein. As gibel carp has no stomach, its digestion and absorption processes are mainly carried out in the intestine. The proteases in fish are mainly synthesized in the liver and exist in the pancreas in the form of the zymogen and then are secreted into the intestine and to be activated into active trypsin which continues to activate other proteases such as chymotrypsin [42, 43]. The present trial showed that the 100% replacement of dietary FM by BLEND B protein increased intestinal TRY activity. Moreover, it was found by liver transcriptome analysis that the protease-related genes were upregulated at dietary 100% replacement of FM by BLEND B. The higher protease activity indicated that absorbable FAAs and polypeptides could be more easily obtained in the intestinal lumen.

The FAAs and peptides in the lumen are absorbed by intestinal epithelial cells through the action of nutrient transporters located on the brush border membrane [44]. The present trial showed that the mRNA expression of *snat2*, *y*^*+*^*lat2*, and *pept1* was upregulated when dietary replacement FM by BLEND B to a certain level respectively, which was beneficial to the absorption of protein by fish. In addition, intestinal morphology (such as villus height, crypt depth, the ratio of villus height to crypt depth, and microvillus length) are important indicators for evaluating intestinal absorptive function [45, 46]. It was found that when dietary 100% replacement FM by BLEND B, the crypt depth became shallower, the villus height to crypt depth ratio (V/C) became larger, and the ML became longer in fish intestines. The higher Vh, the shallower Cd, the larger V/C, and the longer ML in the intestine indicating greater ability of absorption [45–47]. It was also found that dietary 100% replacement FM by BLEND A reduced the Vh and V/C. Similarly, dietary excessive single protein CAP was reported to reduce the Vh of fish intestines [16, 48]. The decreased Vh and V/C implied reduced nutrient absorption and growth retardation [49]. Consistently, dietary 100% replacement FM by BLEND A reduced plasma FAA content. It suggested proper dietary blended protein could improve the digestion and absorption of proteins in gibel carp, which were founded in increased ADC_CP_.

The digestion and absorption of nutrients in the intestine of fish are closely related to health function [50, 51]. On the one hand, inflammation is an important component of intestinal health, and cytokines are pivotal factors mediating inflammatory responses [52]. This study showed that dietary 100% replacement of FM by blended proteins upregulated the mRNA expression of intestinal proinflammatory cytokines (*tnf-α*, *il-6α*, *il-8*, and/or *il-1β*). CPC, used as a single protein to replace dietary FM, was reported to increase the mRNA expression of intestinal proinflammatory cytokines in hybrid grouper (*Epinephelus fuscoguttatus* ♀ × *Epinephelus lanceolatus* ♂) [53, 54]. It was found in the present study that the mRNA expression of *il-10* was upregulated with dietary 100% replacement FM by BLEND B and the mRNA expression of *tgf-β* was upregulated with dietary 100% replacement FM by BLEND C. The upregulation of both proinflammatory and anti-inflammatory cytokines strengthens the immunity of fish [55]. CM and chitin in TMM have been proven to have immunomodulatory influences in fish [21, 56]. However, at dietary 100% replacement of FM by BLEND A, the mRNA expression of *il-10* was significantly downregulated, and intestinal tissue damage occurred, showing a large number of lymphocytes infiltrated into the epithelial cells and the Vh became shorter. This suggested that the inflammation development of the intestine of gibel carp was not conducive to protein digestion and absorption. Similarly, dietary high single protein CAP was reported to cause intestinal inflammation in yellow croaker (*L. crocea*) [16].

Tight junctions between intestinal epithelial cells are an important part of the formation of physical barriers, mainly including the Occludin family, Claudin family, and ZO family proteins [57]. This study found that dietary replacement of FM by blended proteins upregulated the mRNA expression of tight junction-related genes and improved the tight junction damage. It was reported that the upregulation of tight junction-related gene expression favored the integrity of the intestinal epithelium [58, 59]. It suggested that dietary blended proteins of inappropriate ratios could lead to intestinal inflammation of gibel carp. However, intestinal immunity and physical barrier could be improved with proper composition of blend proteins.

Fish intestinal amino acid sensory receptors were reported to be located on the intestinal brush border membrane, mainly including CaSR, T1R1/T1R3, GPRC6A, and mGluR4 [60–62]. This study found that the substitution of FM by blended proteins upregulated the mRNA expression of intestinal amino acid sensory receptor-related genes in gibel carp. The amino acid sensory receptors were mainly reported in mammals that could sense the presence of amino acids in the intestinal lumen and convey the expected signal that nutrients are about to be imminently digested and systemic increase, which is conducive to mobilizing the amino acid metabolism [63, 64]. And similar amino acid sensing mechanism was reported in fish intestines [62, 65]. It suggested that dietary blended proteins enhanced the intestine's sense of amino acids.

## 5. Conclusion

Dietary 100% replacement of FM by the blended TMM, CM, CAP, and CPC at a ratio of 1 : 1:6 : 4 could improve intestine health and protein digestibility, and growth of gibel carp. Dietary DEAA profiles were an important factor affecting the growth, but not EAA profiles. The dietary digestible essential amino acids index (DEEAI) was found as an important indicator of dietary protein quality and should be higher than 79.5% to meet the maximum growth of gibel carp.

## Figures and Tables

**Table 1:** Ingredient (% of dry matter) and chemical composition (% of dry matter) of the experimental diets.

Blended ratios (TMM: CM: CAP: CPC)		1 : 1:8 : 2 (A)			1 : 1:6 : 4 (B)			1 : 1:4 : 6 (C)		
Level of FM replacement (%)	0	33	67	100	33	67	100	33	67	100
Diets	Control	A33	A67	A100	B33	B67	B100	C33	C67	C100
Ingredient										
^1^Fish meal	15.00	10.00	5.00	0.00	10.00	5.00	0.00	10.00	5.00	0.00
^2^*T. molitor* meal	0.00	0.38	0.76	1.14	0.40	0.80	1.20	0.42	0.84	1.26
^3^*Chlorella* meal	0.00	0.38	0.76	1.14	0.40	0.80	1.20	0.42	0.84	1.26
^4^*Clostridium autoethanogenum* protein	0.00	3.04	6.08	9.12	2.40	4.80	7.20	1.68	3.36	5.04
^5^Cottonseed protein concentrated	0.00	0.76	1.52	2.28	1.60	3.20	4.80	2.52	5.04	7.56
^6^Rapeseed meal	25.00	25.00	25.00	25.00	25.00	25.00	25.00	25.00	25.00	25.00
^7^Soybean meal	26.00	26.00	26.00	26.00	26.00	26.00	26.00	26.00	26.00	26.00
Corn starch	15.00	15.00	15.00	15.00	15.00	15.00	15.00	15.00	15.00	15.00
Microcrystalline cellulose	4.38	4.42	4.56	4.70	4.18	4.08	3.98	3.94	3.60	3.26
Fish oil	3.00	3.20	3.35	3.50	3.20	3.35	3.50	3.20	3.35	3.50
Soybean oil	3.00	3.20	3.35	3.50	3.20	3.35	3.50	3.20	3.35	3.50
^8^Mineral premixes	5.00	5.00	5.00	5.00	5.00	5.00	5.00	5.00	5.00	5.00
^9^Vitamin premixes	0.39	0.39	0.39	0.39	0.39	0.39	0.39	0.39	0.39	0.39
Sodium benzoate	0.02	0.02	0.02	0.02	0.02	0.02	0.02	0.02	0.02	0.02
Sodium carboxymethyl cellulose	3.00	3.00	3.00	3.00	3.00	3.00	3.00	3.00	3.00	3.00
Choline chloride	0.11	0.11	0.11	0.11	0.11	0.11	0.11	0.11	0.11	0.11
Yttrium trioxide	0.10	0.10	0.10	0.10	0.10	0.10	0.10	0.10	0.10	0.10
Chemical composition										
Crude protein	34.27	33.81	34.29	34.18	34.36	34.23	34.01	34.09	33.94	33.48
Crude fat	7.99	7.17	7.69	7.81	6.88	7.62	6.82	7.34	6.75	7.25
Ash	10.58	9.69	8.83	9.52	8.5	8.78	8.43	9.66	9.11	8.76
Gross energy (MJ/kg)	18.02	18.23	18.41	18.46	18.39	18.21	18.28	18.38	18.53	17.94

^1^Fish meal: TASA Fish Product Co. Ltd., Peru. ^2^*Tenebrio. molitor* meal: Guangdong Zehecheng Biotechnology Co., Ltd., Guangzhou, China. ^3^*Chlorella* meal: Demeter Bio-Tech Co., Ltd., Wuhan, Hubei, China. ^4^*Clostridium autoethanogenum* protein: Hebei Shoulang Novel Energy Technology Co., Ltd. ^5^Cottonseed protein concentrated: Xinjiang Jinlan Plant Protein Co., Ltd., Xinjiang, China. ^6^Rapeseed meal: Wuhan Coland Feed Co. Ltd., Wuhan, Hubei, China. ^7^Soybean meal: Wuhan Coland Feed Co. Ltd., Wuhan, Hubei, China. ^8^Mineral premixes (mg/kg diet): NaCl, 500.0; MgSO_4_ · 7H_2_O, 8,155.6; NaH_2_PO_4_ · 2H_2_O, 12,500.0; KH_2_PO_4_, 1,6000.0; CaHPO_4_ · 2H_2_O, 7,650.6; FeSO_4_ · 7H_2_O, 2,286.2; C_6_H_10_CaO_6_ · 5H_2_O, 1,750.0; ZnSO_4_ · 7H_2_O, 178.0; MnSO_4_·H_2_O, 61.4; CuSO_4_ · 5H_2_O, 15.5; CoSO_4_ · 7H_2_O, 0.9; KI, 1.5; Na_2_SeO_3_, 0.6; Corn starch, 899.7. ^9^Vitamin premixes (mg/kg diet): Vitamin B_1_, 20; Vitamin B_2_, 20; Vitamin B_6_, 20; Vitamin B_12_, 0.02; folic acid, 5; calcium pantothenate, 50; inositol, 100; niacin, 100; biotin, 0.1; cellulose, 3522; Vitamin A, 11; Vitamin D, 2; Vitamin E, 100; Vitamin K, 10.

**Table 2:** Amino acid (AA, % of dry matter) composition of the experimental diets.

Diets	Control	A33	A67	A100	B33	B67	B100	C33	C67	C100	Requirements
Essential amino acids											
Lysine	2.16	2.15	2.20	2.26	2.20	2.18	2.23	2.13	2.10	2.11	^*∗*^3.30
Methionine	0.56	0.62	0.59	0.57	0.57	0.56	0.58	0.54	0.52	0.49	^#^0.89
Threonine	1.31	1.32	1.35	1.35	1.35	1.34	1.34	1.32	1.31	1.33	^&^1.70
Arginine	1.96	1.96	1.97	2.07	1.92	1.98	2.05	1.99	2.00	2.08	^‼^1.50
Leucine	2.49	2.45	2.46	2.45	2.53	2.55	2.55	2.48	2.43	2.51	^&^1.80
Isoleucine	1.46	1.48	1.47	1.45	1.43	1.49	1.46	1.41	1.41	1.49	^&^1.30
Valine	1.55	1.54	1.52	1.53	1.59	1.58	1.55	1.51	1.55	1.60	^&^1.70
Phenylalanine	1.48	1.51	1.45	1.49	1.57	1.50	1.52	1.45	1.47	1.57	^§^1.10
Histidine	0.69	0.69	0.76	0.73	0.73	0.74	0.63	0.81	0.73	0.76	^§^0.80
EAAI	95.83	97.07	97.52	97.71	97.78	97.86	97.50	96.34	94.86	97.26	—
DEAAI	79.07	82.39	79.22	78.71	81.42	83.19	83.75	80.39	80.15	81.66	—
Nonessential amino acids											
Asparagine	2.77	2.73	2.76	2.73	2.83	2.76	2.82	2.83	2.77	2.84	—
Serine	1.29	1.33	1.36	1.31	1.31	1.31	1.35	1.34	1.33	1.32	—
Glutamic acid	5.57	5.58	5.55	5.61	5.54	5.54	5.68	5.60	5.60	5.65	—
Glycine	1.45	1.43	1.46	1.47	1.47	1.43	1.42	1.51	1.51	1.45	—
Alanine	1.57	1.60	1.63	1.63	1.56	1.59	1.61	1.61	1.62	1.58	—
Tyrosine	0.99	1.05	1.06	1.00	1.00	1.02	1.03	1.01	0.97	1.04	—
Proline	1.55	1.65	1.62	1.56	1.69	1.67	1.98	1.67	1.59	1.74	—
Cysteine	2.21	1.71	1.71	2.09	1.61	1.79	1.71	1.71	1.90	2.12	—

Essential amino acid index (EAAI, %) = 100 × $\sqrt[{n}]{\frac{a}{ar}\times \frac{b}{br}\times \frac{c}{cr}\times \cdot \cdot \cdot \cdot \cdot \cdot \cdot \cdot \cdot \frac{i}{ir}}$. Digestible essential amino acid index (DEAAI, %) = 100 × $\sqrt[{n}]{\frac{A}{ar}\times \frac{B}{br}\times \frac{C}{cr}\times \cdot \cdot \cdot \cdot \cdot \cdot \cdot \cdot \cdot \frac{I}{ir}}$. (*a*, *b*, *c*,…, *i* are the contents of lysine, methionine, threonine, arginine, leucine, isoleucine, valine, phenylalanine, and histidine in each experimental diet; *A*, *B*, *C*,…, *I* are the digestible contents of lysine, methionine, threonine, arginine, leucine, isoleucine, valine, phenylalanine, and histidine in each experimental diet; *ar*, *br*, *cr*,…., *ir* are the requirements of lysine, methionine, threonine, arginine, leucine, isoleucine, valine, phenylalanine, and histidine in gibel carp. *n* is the number of amino acids used).  ^*∗*^Ref from Zhou et al. [23]. ^#^Ref from Jia et al. [24]. ^&^Ref from Li [25]. ^!!^Ref from Tu et al. [26]. ^§^Ref from Ma [27].

**Table 3:** Primer sequences were used for the analysis of mRNA expression by qRT-PCR.

Gene name	GenBank accession number	Forward primer (5′ to 3′)	Reverse primer (5′ to 3′)
*β-actin*	JN006052.1	TGGGACAGAAGGACAGCTATG	AGCTCGTTGTAGAAGGTGTGA
*tnf-α*	XM_026282152.1	TGTTCTCAGGGCATTCGCTT	GGAGTTGTAGTGCCCTTGGT
*il-1β*	XM_026220359.1	GAATGGAAACGACAGCCTCC	GGATTCGTTCAGTTGGCCTC
*il-6α*	XM_026252884.1	GAGATACCGACCACAGCTCA	TGCCCAACTGACTGCATAGA
*il-8*	KC184490.1	CACAAGTGTCGAGCAACCAG	TCAGTTTCAATGCAGCGACA
*il-10*	HQ259106	TGAAAAGGAACGATGGGCAG	TGGAATGATGACGTGCAAGC
*tgf-β*	EU086521.1	GGTTCTTGCGCTGTATAGGC	CCGGCCCACATAGTAAAGGA
*zo-2*	XM_026269460.1	ATGCGTCTGGGAATTACGGG	CATTCCTGAGCCCTTTCCCT
*occludin-a*	HQ110086.1	GGACCAGATCAACAAGCGTC	TTGATGTGGCTGAGTTTGGC
*claudin-b*	HQ656008.1	ACCGGACAGATGCAGTGTAA	ATGATGCCCAGGATCCCAAT
*claudin-c*	HQ656009.1	AGAGTACTGGACAGACGCAG	GATCATCACACGGGCTTTGG
*claudin-d*	HQ656010.1	ACTGCCACAAGATCTCCAGG	GTCCCGTTCTTCAATGCAGT
*claudin-e*	HQ656011.1	AGAAAGCAAGGCAAAGGTGG	TCCCTGACGATGGTGTTAGT
*claudin-h*	HQ656012.1	ACGGCACAAGTAATCTGGGA	CCAAGATGACGGCAATGACC
*y* ^*+*^ *lat2*	XM_026266738.1	ATCATCACTGGCCTGGTCAA	CTGTGACAATGGGCATGGAG
*snat2*	XM_026209285.1	TCACGATCAACACCGAGTCA	ACAGCCCAAATGTGCGAAAT
*pept1*	XM_026265622.1	CCGTACTCATCCTCCCCATC	TCTCGGTCTCTCCTTCCTCA
*casr*	AB713518.1	AACTCCTGGTCTAACGGCAA	AACACCCAACACGAAAGCTG
*gprc6a*	XM_026283875.1	ACGCTGTGTGTTTCATGCAT	GCAAACGATCACATACGGCT
*t1r3*	XM_026275959.1	TTCTGAGCAGCTGGAGAACA	CTCCACTGGACAACGCAAAA
*t1r1*	XM_026268818.1	TGAATGGTCTGATGAGGGCA	GTAAACACATGCTGCCACCA
*mglur4*	EU147495.1	CCAGTATCAGCACGACCTCT	AATCGGCGTGTCATTGTAGC

*β-actin*, reference gene; *tnf-α*, tumor necrosis factor *α*; *il-1β*, interleukin 1*β*; *il-6α*, interleukin 6*α*; *il-8*, interleukin 8; *il-10*, interleukin 10; *tgf-β*, transforming growth factor *β*; *zo-2*, tight junction protein ZO 2; *y*^*+*^*lat2*, Y^+^L amino acid transporter 2-like; *snat2*, sodium-coupled neutral amino acid transporter 2; *pept1*, antigen peptide transporter 1-like; *casr*, extracellular calcium-sensing receptor-like; *gprc6a*, G protein-coupled receptor, class C, group 6, member A; *t1r3*, taste receptor type 1 member 3-like; *t1r1*, taste receptor type 1 member 1-like; *mglur4*, glutamate receptor, metabotropic 4.

**Table 4:** Growth performance and feed utilization indices of gibel carp fed the experimental diets.

Diets	^1^IBW (g)	^2^SR (%)	^3^FR (%BW/d)	^4^FBW (g)	^5^SGR (%/d)	^6^FE (%)	^7^PER	^8^PRE (%)
Control	15.21 ± 0.05	96.20 ± 1.71	3.03 ± 0.03	59.05 ± 0.42^bc^	2.42 ± 0.01^bc^	65.42 ± 1.43^b^	1.91 ± 0.04^b^	33.67 ± 0.68^c^
A33	15.15 ± 0.07	96.67 ± 1.71	2.94 ± 0.03	61.38 ± 0.68^ab^	2.50 ± 0.02^ab^	69.74 ± 0.82^a^	2.06 ± 0.02^a^	35.54 ± 0.46^ab^
A67	15.20 ± 0.10	97.60 ± 0.50	3.00 ± 0.05	56.91 ± 0.67^c^	2.36 ± 0.02^c^	65.90 ± 1.05^ab^	1.92 ± 0.03^b^	33.90 ± 0.87^bc^
A100	15.18 ± 0.10	96.20 ± 2.06	3.06 ± 0.03	53.68 ± 1.02^d^	2.25 ± 0.04^d^	61.79 ± 0.99^c^	1.81 ± 0.03^c^	31.52 ± 0.41^d^
B33	15.20 ± 0.06	95.23 ± 3.42	2.98 ± 0.04	60.53 ± 0.60^ab^	2.47 ± 0.01^ab^	67.15 ± 0.86^ab^	1.95 ± 0.03^ab^	34.76 ± 0.71^abc^
B67	15.23 ± 0.12	96.67 ± 1.26	2.95 ± 0.02	60.21 ± 0.81^ab^	2.45 ± 0.02^ab^	68.30 ± 1.30^ab^	1.99 ± 0.04^ab^	35.52 ± 0.49^ab^
B100	15.15 ± 0.08	96.67 ± 1.71	2.94 ± 0.03	61.81 ± 1.07^a^	2.51 ± 0.03^a^	69.75 ± 0.85^a^	2.06 ± 0.03^a^	36.33 ± 0.24^a^
C33	15.07 ± 0.05	95.70 ± 2.48	2.95 ± 0.04	60.25 ± 0.70^ab^	2.48 ± 0.02^ab^	68.23 ± 1.96^ab^	2.00 ± 0.06^ab^	34.14 ± 0.50^bc^
C67	15.20 ± 0.03	95.70 ± 0.81	2.94 ± 0.05	58.98 ± 0.66^bc^	2.42 ± 0.02^bc^	67.61 ± 1.11^ab^	1.99 ± 0.03^ab^	34.44 ± 0.20^bc^
C100	15.26 ± 0.04	96.67 ± 0.97	2.97 ± 0.02	59.05 ± 0.41^bc^	2.42 ± 0.01^bc^	66.82 ± 0.64^ab^	1.98 ± 0.02^ab^	35.25 ± 0.36^abc^

Values are means ± SE (*n* = 3). Values in the same column with different letters a, b, and c are significantly different (*P* < 0.05). ^1^IBW, Initial body weight (g). ^2^SR, Survival rate (%) = 100 × (final fish number/initial fish number). ^3^FR, Feeding rate (%BW/d) = 100 × intake of feed dry matter/(days × (final body weight + initial body weight)/2). ^4^FBW, Final body weight (g). ^5^SGR, Specific growth rate (%/day) = 100 × (ln final body weight−ln initial body weight)/days. ^6^FE, Feed efficiency (%) = 100 × (weight gain + dead fish weight)/dry weight of feed. ^7^PER, Protein efficiency ratio = (final body weight−initial body weight)/intake of protein. ^8^PRE, Protein retention efficiency (%) = 100 × amount of body protein deposition/intake of protein.

**Table 5:** Body indexes and proximate composition of whole body of gibel carp fed the experimental diets.

Diets	Body indexes			Proximate composition of whole body (%)			
	^1^CF (g/cm^3^)	^2^VSI (%)	^3^HSI (%)	Moisture	Ash	Crude protein	Crude fat
Control	3.14 ± 0.06	11.57 ± 0.37	2.87 ± 0.20^b^	71.18 ± 0.45	4.27 ± 0.08	16.93 ± 0.22	6.20 ± 0.39
A33	3.30 ± 0.10	13.06 ± 0.54	4.30 ± 0.47^a^	70.74 ± 0.09	4.40 ± 0.04	16.73 ± 0.17	6.39 ± 0.31
A67	3.17 ± 0.05	12.06 ± 0.53	3.02 ± 0.38^b^	71.81 ± 0.23	4.48 ± 0.02	16.92 ± 0.16	5.34 ± 0.39
A100	3.14 ± 0.07	12.20 ± 0.36	2.98 ± 0.17^b^	71.64 ± 0.57	4.45 ± 0.08	16.79 ± 0.28	5.67 ± 0.11
B33	3.24 ± 0.08	12.90 ± 0.66	3.15 ± 0.17^ab^	71.05 ± 1.25	4.35 ± 0.09	16.79 ± 0.35	5.96 ± 0.86
B67	3.24 ± 0.07	13.09 ± 0.64	3.52 ± 0.35^ab^	71.39 ± 1.16	4.38 ± 0.02	16.79 ± 0.37	5.72 ± 1.02
B100	3.29 ± 0.07	12.31 ± 0.61	3.46 ± 0.51^ab^	71.39 ± 0.67	4.27 ± 0.07	16.69 ± 0.31	5.95 ± 0.79
C33	3.34 ± 0.07	12.43 ± 0.52	3.47 ± 0.35^ab^	71.24 ± 0.39	4.24 ± 0.03	16.20 ± 0.24	6.40 ± 0.41
C67	3.34 ± 0.07	11.83 ± 0.45	3.06 ± 0.36^b^	70.96 ± 1.22	4.36 ± 0.11	16.50 ± 0.29	6.03 ± 0.64
C100	3.33 ± 0.06	13.20 ± 0.56	3.19 ± 0.55^ab^	71.44 ± 0.49	4.39 ± 0.06	17.15 ± 0.12	5.52 ± 0.22

Values are means ± SE (*n* = 3 for proximate composition of whole body, *n* > 6 for body indexes). Values in the same column with different letters a and b are significantly different (*P* < 0.05). ^1^CF, Condition factor (g/cm^3^) = 100 × body weight/(total length)^3^. ^2^VSI, Visceral somatic index (%) = 100 × (visceral weight/body weight). ^3^HSI, Hepatosomatic index (%) = 100 × (liver weight/body weight).

**Table 6:** The plasma biochemical indices of gibel carp fed the experimental diets.

Diets	TG (mg/dL)	CHO (mg/dL)	GLU (mg/dL)	FAA (*μ*mol/mL)
Control	115.66 ± 11.68	284.38 ± 7.05^ab^	47.59 ± 5.21^ab^	86.19 ± 3.61^c^
A33	111.19 ± 11.40	295.83 ± 15.18^ab^	46.72 ± 5.66^ab^	108.33 ± 6.95^ab^
A67	141.43 ± 9.61	268.54 ± 13.97^ab^	51.98 ± 5.17^a^	87.62 ± 4.92^c^
A100	136.00 ± 16.86	254.17 ± 12.06^b^	47.62 ± 4.50^ab^	66.90 ± 2.51^d^
B33	141.75 ± 12.68	272.50 ± 14.63^ab^	30.48 ± 2.57^c^	94.52 ± 8.80^bc^
B67	127.80 ± 12.23	254.17 ± 12.58^b^	38.46 ± 5.08^abc^	115.48 ± 6.76^a^
B100	133.42 ± 23.81	263.33 ± 9.29^ab^	33.60 ± 3.50^bc^	95.24 ± 5.78^bc^
C33	126.42 ± 17.88	296.88 ± 16.56^a^	30.45 ± 6.59^c^	90.00 ± 5.79^bc^
C67	103.84 ± 16.24	254.58 ± 12.70^b^	30.60 ± 4.23^a^	86.90 ± 5.53^c^
C100	130.61 ± 26.32	271.25 ± 9.59^ab^	27.92 ± 3.40^a^	90.64 ± 6.02^bc^

Values are means ± SE (*n* = 6). Values in the same column with different letters a, b, and c are significantly different (*P* < 0.05). TG, triglycerides; CHO, cholesterol; GLU, glucose; FAA, free amino acid.

**Table 7:** Apparent digestibility coefficients of substance (%) of gibel carp fed the experimental diets.

Diets	Control	A33	A67	A100	B33	B67	B100	C33	C67	C100
Dry matter	55.96 ± 0.70^bc^	57.46 ± 0.83^ab^	49.75 ± 0.88^e^	49.40 ± 1.13^e^	52.53 ± 0.83^d^	55.10 ± 0.72^bcd^	58.88 ± 0.75^a^	58.80 ± 0.78^a^	55.87 ± 0.58^bc^	54.05 ± 1.06^cd^
Gross energy	61.15 ± 0.78^ab^	62.16 ± 1.13^a^	53.46 ± 0.79^e^	53.73 ± 1.05^e^	56.95 ± 0.74^d^	57.41 ± 0.84^cd^	60.09 ± 0.83^abc^	62.66 ± 1.02^a^	59.06 ± 0.68^bcd^	54.17 ± 0.81^e^
Crude protein	78.01 ± 0.41^c^	82.68 ± 0.42^a^	76.06 ± 0.58^d^	72.50 ± 0.46^e^	79.35 ± 0.47^b^	79.38 ± 0.53^b^	82.25 ± 0.37^a^	79.45 ± 0.32^b^	78.40 ± 0.02^bc^	79.19 ± 0.35^bc^
Total essential amino acid	84.58 ± 0.32^d^	86.70 ± 0.33^c^	83.37 ± 0.24^e^	82.93 ± 0.33^e^	85.24 ± 0.40^d^	86.79 ± 0.12^bc^	87.60 ± 0.10^b^	85.31 ± 0.07^d^	86.37 ± 0.28^c^	85.44 ± 0.55^d^
Total non-essential amino acid	83.15 ± 0.43^d^	85.13 ± 0.27^bc^	82.29 ± 0.37^e^	78.61 ± 0.19^f^	82.88 ± 0.13^de^	84.86 ± 0.16^bc^	87.69 ± 0.11^a^	83.22 ± 0.40^d^	85.62 ± 0.23^b^	84.63 ± 0.10^c^
Essential amino acids										
Lysine	84.20 ± 0.60^dc^	86.74 ± 0.33^a^	84.10 ± 0.40^dc^	83.16 ± 0.46^d^	85.55 ± 0.28^ab^	85.58 ± 0.38^ab^	86.70 ± 0.28^a^	84.39 ± 0.37^bcd^	85.12 ± 0.36^bc^	83.96 ± 0.28^dc^
Methionine	97.08 ± 0.19^b^	97.79 ± 0.59^ab^	97.07 ± 0.57^b^	96.79 ± 0.04^b^	97.57 ± 0.07^ab^	97.75 ± 0.08^ab^	98.24 ± 0.10^a^	97.54 ± 0.57^ab^	97.7 ± 0.06^ab^	97.79 ± 0.08^ab^
Threonine	75.19 ± 0.79^e^	77.77 ± 0.34^ab^	75.61 ± 0.47^de^	71.68 ± 0.38^f^	76.84 ± 0.28^bcd^	77.44 ± 0.29^bc^	78.92 ± 0.41^a^	76.11 ± 0.52^cde^	76.53 ± 0.35^bcde^	76.62 ± 0.54^bcde^
Arginine	76.74 ± 1.73^bc^	76.11 ± 0.82^bcd^	71.15 ± 0.77^f^	72.69 ± 0.97^def^	72.44 ± 0.95^ef^	80.88 ± 0.50^a^	78.84 ± 1.45^ab^	76.26 ± 1.29^bc^	79.05 ± 0.80^ab^	75.10 ± 1.22^cde^
Leucine	82.75 ± 0.42^e^	85.81 ± 0.45^b^	82.21 ± 0.31^ef^	81.36 ± 0.35^f^	83.93 ± 0.32^d^	85.27 ± 0.32^bc^	86.97 ± 0.37^a^	84.38 ± 0.14^cd^	85.26 ± 0.26^bc^	84.52 ± 0.22^bc^
Isoleucine	82.32 ± 0.02^d^	86.94 ± 0.42^a^	83.39 ± 0.54^d^	82.48 ± 0.33^d^	84.76 ± 0.27^bc^	84.66 ± 0.65^bc^	85.77 ± 0.05^b^	85.24 ± 0.22^bc^	84.46 ± 0.19^c^	85.62 ± 0.23^b^
Valine	79.69 ± 0.20^cd^	83.02 ± 0.55^a^	75.74 ± 0.71^e^	75.27 ± 0.50^e^	81.01 ± 0.86^bc^	82.53 ± 0.49^ab^	83.30 ± 0.39^a^	78.87 ± 0.63^cd^	82.02 ± 0.31^ab^	82.31 ± 0.28^ab^
Phenylalanine	82.31 ± 0.77^cde^	84.66 ± 0.85^ab^	77.41 ± 0.37^f^	79.99 ± 0.94^ef^	82.68 ± 1.77^cde^	84.65 ± 0.37^ab^	87.65 ± 0.35^a^	81.17 ± 1.36^ed^	84.34 ± 0.91^cd^	83.51 ± 1.18^cd^
Histidine	84.11 ± 0.90^b^	86.78 ± 1.56^ab^	87.24 ± 0.46^a^	84.28 ± 1.12^b^	86.90 ± 0.47^ab^	87.72 ± 0.84^a^	88.18 ± 0.82^a^	89.20 ± 1.29^a^	87.60 ± 0.52^a^	88.24 ± 0.16^a^
Non-essential amino acids										
Asparagine	82.13 ± 0.64^b^	85.21 ± 0.29^d^	81.64 ± 0.41^b^	78.28 ± 0.05^a^	83.80 ± 0.19^c^	84.37 ± 0.33^cd^	87.38 ± 0.45^e^	83.78 ± 0.47^c^	84.33 ± 0.30^cd^	85.03 ± 0.22^d^
Serine	80.37 ± 0.56^c^	83.49 ± 0.34^ab^	82.6 ± 0.44^b^	79.23 ± 0.38^b^	83.07 ± 0.31^ab^	82.68 ± 0.38^b^	84.33 ± 0.31^a^	83.22 ± 0.55^ab^	82.58 ± 0.35^b^	83.76 ± 0.40^ab^
Glutamic acid	88.21 ± 0.79^bc^	89.75 ± 0.29^b^	87.26 ± 0.24^cd^	85.87 ± 0.01^d^	88.56 ± 0.44^bc^	89.52 ± 0.42^b^	91.45 ± 0.42^a^	88.67 ± 0.68^bc^	89.88 ± 0.64^b^	89.33 ± 0.64^b^
Glycine	73.59 ± 0.80^d^	76.92 ± 0.64^c^	76.23 ± 1.06^c^	68.22 ± 0.43^e^	75.18 ± 0.50^cd^	79.08 ± 0.39^b^	84.62 ± 0.45^a^	76.64 ± 0.75^c^	80.83 ± 0.48^b^	79.02 ± 0.45^b^
Alanine	80.33 ± 1.13^b^	81.51 ± 0.39^b^	82.08 ± 0.63^b^	74.72 ± 0.87^c^	80.54 ± 0.76^b^	81.73 ± 1.03^b^	85.84 ± 0.72^a^	81.97 ± 1.75^b^	82.56 ± 0.50^ab^	82.93 ± 1.97^ab^
Tyrosine	83.77 ± 0.59^c^	86.64 ± 0.73^a^	84.58 ± 0.38^bc^	80.43 ± 0.27^d^	83.85 ± 0.94^c^	86.51 ± 0.74^a^	86.83 ± 0.19^a^	84.10 ± 0.25^bc^	85.69 ± 0.64^ab^	85.51 ± 0.29^abc^
Proline	77.77 ± 0.44^d^	80.80 ± 0.59^bc^	74.25 ± 1.74^e^	72.32 ± 0.68^e^	79.05 ± 0.69^cd^	82.35 ± 0.76^b^	88.02 ± 0.62^a^	79.00 ± 0.75^cd^	82.26 ± 0.95^b^	80.24 ± 0.35^bcd^
Cysteine	85.11 ± 1.01^a^	84.80 ± 0.45^ab^	78.65 ± 0.19^e^	73.15 ± 0.37^f^	74.65 ± 0.56^f^	81.62 ± 1.16^cd^	82.73 ± 0.79^bc^	74.79 ± 1.13^f^	86.23 ± 0.47^a^	79.86 ± 0.23^de^

Values are means ± SE (*n* = 3), and values in the same row with different letters a, b, c, d, e, and f are significantly different (*P* < 0.05). Apparent digestibility coefficients of substance (%) = 100 × [1−(fecal substance/dietary substance) × (Y_2_O_3_ in diet/Y_2_O_3_ in feces)].

## Data Availability

Data will be made available on request.
